# Virulence Factors and Antibiotic Resistance of Enterobacterales

**DOI:** 10.3390/microorganisms10081588

**Published:** 2022-08-07

**Authors:** Dobroslava Bujňáková, Nikola Puvača, Ivana Ćirković

**Affiliations:** 1 Institute of Animal Physiology, Centre of Biosciences of the Slovak Academy of Sciences, 04001 Košice, Slovakia; 2 Department of Engineering Management in Biotechnology, Faculty of Economics and Engineering Management in Novi Sad, University Business Academy in Novi Sad, 21000 Novi Sad, Serbia; 3 Faculty of Biomedical and Health Sciences, Jaume I University, Avinguda de Vicent Sos Baynat, s/n, 12071 Castelló de la Plana, Spain; 4 Institute of Microbiology and Immunology, Faculty of Medicine, University of Belgrade, dr Subotica 1, 11000 Belgrade, Serbia

In the class Gammaproteobacteria, Enterobacterales are Gram-negative, facultatively anaerobic bacteria [1]. In 2021, seven validly published families belonged to the order Enterobacterales. This order is characterized by the genus *Enterobacter*. Although it includes many harmless bacteria, the Enterobacterales is an order of bacteria within the Proteobacteria phylum, which is responsible for many enteric pathogens, known as *Salmonella, Shigella*, *Escherichia coli*, *Yersinia pestis, Klebsiella, Shigella, Proteus, Serratia*, and *Citrobacter*. 

In healthy conditions, this order of bacteria constitutes the microbiota’s minor bacterial constituents that permanently colonize the human gut. In the presence of a balanced microbiota and a complex and dense bacterial community, opportunistic Enterobacterales can persist as gut commensals without causing infection. Inflammatory host reactions can be triggered by a bloom of Enterobacterales following disturbed microbiota, which can lead to pathogen-mediated infections. 

The vast majority of bacteria belonging to Enterobacterales harbor features that can confer their virulence and pathogenicity or phenotypes, which may result in severe health concerns, such as multi-drug resistance and/or biofilm production [2]. To improve invasiveness, overcome host defenses, and cause infection, Enterobacterales employ a multitude of strategies. Various strains can use alternative virulence factors with similar functions, with this plasticity resulting in unique combinations between the factors [3]. The significant (most important) classes of virulence factors are adhesins, fimbriae, intimin, capsules, iron metabolism, siderophores, heme/hemoglobin transport protein and receptor, cell invasion, outer membrane porin A, etc.

Detailed information on virulence genes has been provided, particularly on those related to *Klebsiella* spp. and *Escherichia coli*, for which several genes associated with harmful traits were identified [4]. *E. coli* strain is a typical exemplar with regard to the traits that differentiate commensalism and pathogenicity; it is a part of the normal intestinal microbiota but can also cause serious intestinal and extra-intestinal infections in humans and animals [5]. The genetic determinants associated with virulence and invasiveness of other genera, including *Enterobacter*, *Cronobacter*, and *Citrobacter*, are less well understood. 

A major challenge of the twenty-first century involves the emergence of ARB in human and veterinary medicine. By horizontal gene transfer through plasmids, transposons, and integrons, which can integrate or excise gene cassettes, bacteria can become resistant to antibiotics. Even though antibiotic resistance is not in itself a virulence factor, it can play a significant role in infection development in certain varieties of ecological niches that antibiotic-resistant bacteria can colonize.

“Virulence Factors and Antibiotic Resistance of Enterobacterales” gathers valuable research papers related to the Enterobacterales and specific virulent and resistant features of *Salmonella enterica, Escherichia coli, Klebsiella* spp., and *Cronobacter sakazakii.*

More specifically, this Special Issue includes studies investigating the effect of *sdi*A mutation by using the CRISPR-Cas9 system on *S. enterica* virulence and host pathogenesis [6]; the study of the phylogenetic relationships, virulence genes, phenotypes, resistance to antibiotics, and bacterial morphology of uropathogenic *E. coli* [7]; the identification of commensal *Klebsiella* isolates from the milk, meconium, and feces samples compared with nosocomial isolates from an NICU outbreak and from community-acquired infection isolates [8]; the investigation of the *omp*F gene function that encodes outer membrane protein F (OmpF) in *Cronobacter sakazakii* by generating an *omp*F deletion mutant (∆*omp*F) and complementation controls [9]; the characterization of the AST profile of K1-antigenic *E. coli* among hospitalized patients both adults and children [10]; the examination of *E. coli* isolates from unpasteurized ovine cheeses for the possession of traits associated with the virulence of human extra-intestinal pathogenic *E. coli* (ExPEC) or intestinal Vero (Shiga) toxin (Vtx or Stx)-producing *E. coli* (VTEC or STEC), e.g., *iutA, iss, cvaC, kpsII, tsh, papC, fyuA, cnf1, stx1, stx2,* and *eaeA* and their phylogenetic grouping [11]; the comparison of the *E. coli* isolates and plasmids collected from humans, animals, and wildlife to best understand the spread of CTX-M-1 among these ecosystems [12]; the evaluation of the phenotypical and genotypical AMR of diarrheal *E. coli* from companion animals to ascertain biofilm-forming capacity as one of the causes promoting enhanced resistance [13]; detecting septic conditions early by studying the morphology of T-lymphocytes after exposure to bacterial determinants [14]; and finally, pandemic clones of CTX-M-15 producing *K. pneumoniae* ST15, ST147, and ST307 in companion parrots [15].

Altogether, the papers of this Special Issue present valuable data on the virulence, resistance, and biofilm formation of various types of bacteria from Enterobacterales in a range of animals, humans, and ecosystems.
